# Food Intolerance: The Role of Histamine

**DOI:** 10.3390/nu13093207

**Published:** 2021-09-15

**Authors:** Yulia O. Shulpekova, Vladimir M. Nechaev, Irina R. Popova, Tatiana A. Deeva, Arthur T. Kopylov, Kristina A. Malsagova, Anna L. Kaysheva, Vladimir T. Ivashkin

**Affiliations:** 1 Department of Internal Diseases Propedeutics, Sechenov University, 119121 Moscow, Russia; shulpekova_yu_o@staff.sechenov.ru (Y.O.S.); nechaev_v_m@staff.sechenov.ru (V.M.N.); popova_i_r@staff.sechenov.ru (I.R.P.); ivashkin_v_t@staff.sechenov.ru (V.T.I.); 2 Department of Biological Chemistry, Sechenov University, 119991 Moscow, Russia; deeva_t_a@staff.sechenov.ru; 3 Biobanking Group, Branch of Institute of Biomedical Chemistry “Scientific and Education Center”, 123098 Moscow, Russia; a.t.kopylov@gmail.com (A.T.K.); kaysheva1@gmail.com (A.L.K.)

**Keywords:** histamine, food intolerance, irritable bowel syndrome, metabolite, health

## Abstract

Histamine is a natural amine derived from L-histidine. Although it seems that our knowledge about this molecule is wide and diverse, the importance of histamine in many regulatory processes is still enigmatic. The interplay between different types of histamine receptors and the compound may cause ample effects, including histamine intoxication and so-called histamine intolerance or non-allergic food intolerance, leading to disturbances in immune regulation, manifestation of gastroenterological symptoms, and neurological diseases. Most cases of clinical manifestations of histamine intolerance are non-specific due to tissue-specific distribution of different histamine receptors and the lack of reproducible and reliable diagnostic markers. The diagnosis of histamine intolerance is fraught with difficulties, in addition to challenges related to the selection of a proper treatment strategy, the regular course of recovery, and reduced amelioration of chronic symptoms due to inappropriate treatment prescription. Here, we reviewed a history of histamine uptake starting from the current knowledge about its degradation and the prevalence of histamine precursors in daily food, and continuing with the receptor interactions after entering and the impacts on the immune, central nervous, and gastrointestinal systems. The purpose of this review is to build an extraordinarily specific method of histamine cycle assessment in regard to non-allergic intolerance and its possible dire consequences that can be suffered.

## 1. Introduction

Histamine is a biogenic amine with a wide range of biological effects on various types of cells. These effects are mediated by the activation of four subtypes of histamine receptors—H1R, H2R, H3R, and H4R [1]. The signaling mechanisms are different for each type of receptor; thus, H1R receptors are associated with the breakdown of phosphoinositides and calcium mobilization [2], while H2 receptors are associated with adenylate cyclase [3] and H3R is mediated by interactions with Gαi/o stimulation, which reduces the production and release of neurotransmitters [4]. Stimulation of H4R reduces forskolin-induced cyclic AMP formation, which leads to the activation of MAPK and enhanced Ca^2+^ release [5].

The biological effect of histamine depends on the receptor subtype and cell type. In the gastrointestinal tract, histamine is present in relatively high concentrations, especially during inflammatory reactions. This biogenic amine acts on a variety of cell types, including smooth muscle cells, neurons, endocrine and exocrine cells, blood cells, and cells of the immune system [6]. In scientific research, much attention is paid to the role of histamine in the immune response in allergic inflammation and anaphylaxis. Allergic diseases such as allergic asthma, pruritus, atopic dermatitis, and allergic rhinitis arise from complex interactions between inflammatory cells, including mast cells, basophils, lymphocytes, dendritic cells, neutrophils, and eosinophils, in response to various environmental factors [7,8]. These cells produce a variety of inflammatory mediators such as histamine, eicosanoids, chemokines, cytokines, and reactive oxygen species [9]. Among them, the mast cell histamine is a pivotal player in stimulating the development of inflammatory diseases associated with allergies by regulating the maturation and activation of leukocytes and directing their migration to target sites, where they induce chronic inflammation [10,11]. Histamine also performs various other immunoregulatory functions by modulating the functions of monocytes [12], T cells [13], macrophages [14], neutrophils [15], and other immune response cells [8].

More attention is paid to the role of histamine in physiological processes such as histamine intoxication, a syndrome of histamine intolerance [1,16].

Histamine intolerance arises from the imbalance of accumulated histamine and the ability to degrade histamine [17]. In healthy people, exogenous or dietary histamine is eliminated primarily with the participation of intestinal diamine oxidases (DAOs) [18]. Insufficient expression of secretory DAO (due to genetic polymorphisms) or inhibition of their activity (xenobiotics or drugs) increases the risk of histamine toxicity to the human body [19,20]. Another pathway for the elimination of histamine with the participation of histamine-N-methyltransferase is a cytosolic protein that can convert histamine only in the intracellular space of cells. Disruption of histamine degradation based on decreased DAO activity and the resulting excess histamine can cause numerous symptoms that mimic an allergic reaction. This article discusses some of the biological effects. We discuss the effects of exogenous histamine on gastrointestinal disorders, including food allergies, scombroid food poisoning, histamine intolerance, irritable bowel syndrome, and inflammatory bowel disease [1]. We consider the probable role of mast cells as the main source of endogenous histamine in the realization of innate and acquired immunity reactions.

## 2. Metabolism of Histamine

Histamine (2-[4-imidazole]-ethylamine) has important regulatory functions. This biogenic amine is formed from L-histidine in almost all organs and tissues using L-histidine decarboxylase, with vitamin B6 (pyridoxal phosphate) being a co-factor. Approximately 5% of the total amount of histamine enters the body ready-made with food and is synthesized by intestinal microorganisms (the so-called exogenous histamine). Bacterial and human decarboxylases are activated under the influence of an increased demand for histamine; for example, it is necessary to regulate the intraplasmic pH under conditions of lactic acidosis [21,22].

In cells, histamine is concentrated mainly in the microsomes and the nucleus. Nuclear chromatin has the highest affinity for histamine; however, this affinity decreases with active DNA division during regeneration [22]. The main depot cells of histamine are basophils and mast cells in the endoplasmic granules, which accumulate in significant amounts (>90% of the total intracellular pool). The regulatory role of histamine in the cell is not well understood; therefore, pharmacological intervention that affects its intracellular metabolism can lead to unpredictable consequences [23].

**Figure 1:**
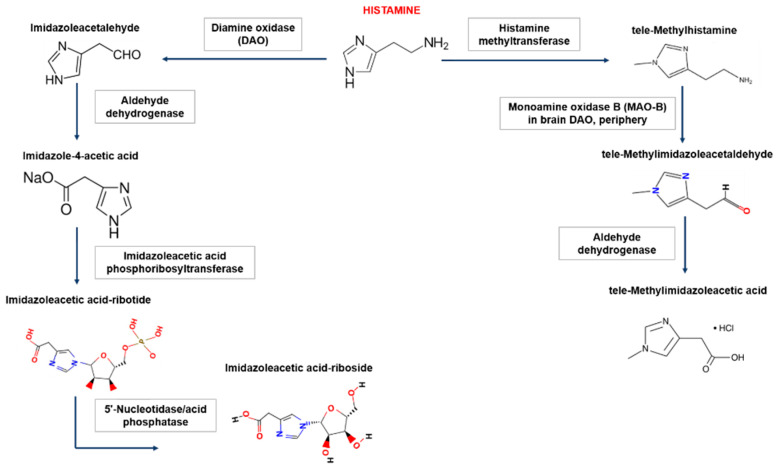
Biogenic amine histamine metabolism. In the human body, histamine is metabolized in two ways: (1) extracellular oxidative deamination of the primary amino group by the enzyme diamine oxidase (DAO) and (2) intracellular methylation of the imidazole ring by the enzyme histamine N-methyltransferase. Inhibition of enzymes is carried out by reaction products of the type of negative feedback and xenobiotics (drugs). Oxidative deamination of histamine DAO leads to the formation of imidazole acetaldehyde, then via aldehyddehydrogenase, imidazole-4-acetic acid is formed. The latter product, after the attachment of the ribose molecule, forms another form for excretion. The product of histamine methylation, catalyzed by histamine-N-methyltransferase, is tele-methylhistamine, which is subsequently converted by monoamine oxidase to tele-methylimidazole acetaldehyde. The latter is then converted to tele-methylimidazoleacetic acid with the participation of aldehyde dehydrogenase.

In the human body, there are two main pathways for the degradation of histamine, which involve diamine oxidase (DAO, synonym-histaminase) or histamine-*N*-methyltransferase (Figure 1).

DAO is mainly synthesized in the mucous membrane of the small and ascending colon (activity increases in the distal direction), placenta, and kidneys. Accumulating in intracellular vesicles, DAO then enters the extracellular space, where it degrades histamine. DAO performs a “barrier function”, thereby limiting the passage of histamine from the intestines into the blood; however, histamine-*N*-methyltransferase methylates histamine via the participation of B vitamins and S-adenosylmethionine [24]. This enzyme regulates histamine content within the cell and is present in most tissues. The activity of this enzyme is especially high in the kidneys, liver, spleen, colon, lungs, leukocytes, megakaryocytes, prostate gland, ovaries, histaminergic neurons of the spinal cord, hypothalamus, and peripheral neurons. The polymorphism of DAO and histamine-*N*-methyltransferase genes may underlie individual differences in histamine metabolism. In the synapses, acetaldehyde dehydrogenase plays a role in the degradation of histamine [20].

Histamine intolerance, called enteric histaminosis or dietary histamine sensitivity, is a disorder associated with an impairment in the ability to metabolize this metabolite [24]. Food histamine enters the body through the intestinal epithelium. The main role in the metabolism of exogenous (food, microbiota) histamine is played by DAO [18,25,26]. The key role of DAO in histamine metabolism was confirmed in the second half of the 20th century in numerous studies in animal models and in vitro. In animal models, enteral-induced histaminosis was observed under conditions of an excess of histamine in the intestinal lumen, factors that reduce DAO activity in intestinal cells, or a high content of free histamine circulating in the bloodstream [18,25,26,27]. Subsequently, negative symptoms of enteral histominosis have been demonstrated in humans [19,28].

Thus, Sattler and Lorenz [19] confirmed the development of pathological symptoms in pigs with an excess of histamine in the diet. The clinical signs of disease in the test animals were characterized by high biological variability, confirming the danger of even moderate histamine load [19]. In the intestinal lumen, the level of histamine can be increased not only by oral consumption, but also probably as a result of decarboxylation of histidine of hemoglobin during gastrointestinal bleeding, intestinal obstruction, and septic conditions. An important source of histamine is the highly active histidine decarboxylases of the microbiota [29,30]; however, the phenomenon of increased histamine levels in septic conditions is still controversial [31,32].

Using the cecal ligation and puncture (CLP) mouse model, Mizuki Hattori et al. showed an increase in circulating and tissue histamine levels after induction of sepsis and its role as a mediator of damage to the main target organs (lungs, liver, and kidneys) [33]. In addition, the expression level of the H1 receptor gene was increased in the heart, spleen, and intestines, while the expression of the H2 receptor gene was increased in the liver and kidneys. Treatment with a combination of H1 and H2 receptor antagonists significantly reduced serum aminotransferase activity, creatinine levels, and proinflammatory cytokines in CLP mice [33]; hence, reductions in histamine levels may reduce multiple organ damage caused by sepsis; however, the primary secretory origin of histamine during sepsis remains controversial and requires further clinical studies and evaluation [34].

The reason for the excess histamine is the inhibition of DAO by widely used mucolytic (ambroxol, acetylcysteine), antiemetic (metoclopramide), antiarrhythmic (verapamil, prajmalium), and antihypertensive drugs (dihydralazine), as well as antidepressants (amitriptylerline) and other drugs [19,35].

Finally, increased plasma levels of histamine were shown in pig and dog models after intravenous administration of this metabolite [36,37]; however, it is possible to avoid enteral-induced histaminosis by avoiding DAO-blocking drugs [19], a low-histamine diet [38], and the use of antihistamines in the intensive care unit to reduce the likely risk of high plasma histamine levels.

## 3. Histamine Receptors

**Figure 2:**
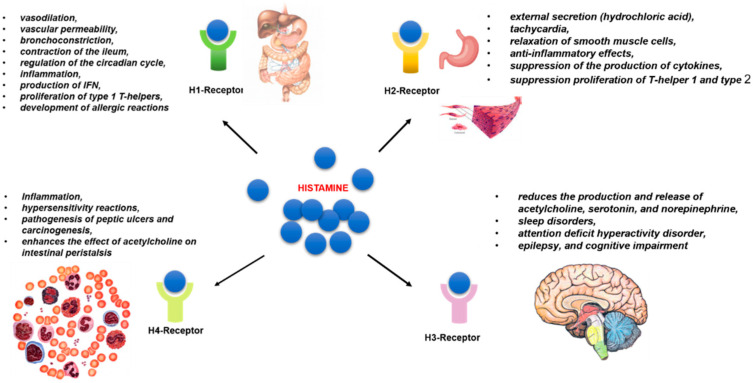
The biological effects of histamine receptors. Histamine of exogenous or endogenous origin is involved in the regulation of a wide range of metabolic transformations by binding to four types of receptors, designated as H1–H4 subtypes. Receptors of the H1 subtype are localized in the GI tract and endoteium and are involved in allergic inflammation and vasodilation, while receptors of the H2 subtype are highly expressed in various cells and tissues, such as B cells, T cells, dendritic cells, and gastric parietal cells. H2 receptors are involved in gastric acid secretion, relaxation of smooth muscle cells, and immune cell differentiation. The H3 receptors are expressed in neurons and play an important role in neurotransmission (neuronal function cognition, regulation of neuronal histamine turnover). The histamine H4R is expressed in a variety of immune cells and is involved in immunomodulation, including immune cell chemotaxis, immune response, and inflammation. The last two subtypes are characterized by high affinity for histamine.

Histamine has a multifaceted effect on metabolic transformations in the human body in health and disease by activating four subtypes of rhodopsin-like G-protein-coupled receptors (GPRC) (H1, H2, H3, and H4) [39]. The receptor subtypes are characterized by varying affinity for the target histamine molecule [40]. Receptors H1 and H2 exhibit low affinity for histamine with a dissociation constant K_d_ ~ μM. While the H3 and H4 subtypes have high affinity, their K_d_ is ~5–10 nM [41]. The genes encoding HR synthesis are subject to alternative splicing. Their activity can vary depending on the homeostatic conditions [42] (Figure 2).

H1Rs are present in the endothelium and vascular smooth muscle cells of almost all tissues (their density is especially high along the gastrointestinal tract), as well as in the hypothalamus, adrenal medulla, and immunocompetent cells. The main effects of H1R stimulation are inflammation, systemic vasodilation, increased vascular permeability, bronchoconstriction, contraction of the ileum, and regulation of the circadian cycle [42]. Allergic symptoms are mainly caused by IgE-mediated activation of mucosal mast cells, which results in the release of histamine and many other mediators [43]. H1R is the main receptor involved in the development of allergic reactions. H1R protein and H1R mRNA are elevated in allergic conditions that aggravates allergic symptoms [16,44]. Non-slow-type hypersensitivity reactions (erythema, pruritus, and edema) can be caused by H1R activation [45]. Histamine activates H1R through the Gαq/11 protein, triggering a cascade of transformations through the activation of phospholipase C and increasing Ca^2+^ evels [46,47]. As a consequence, histamine causes contraction of airway smooth muscle, increases vascular permeability, and induces the production of prostacyclin- and platelet-activating factor [48]. H1R activation has a pro-inflammatory consequence, namely the production of IFN, leading to the proliferation of type 1 T-helpers [49].

A large number of scientific studies have highlighted the combined effects of histamine and H1R cytokines in the development of allergic conditions and inflammation, the so-called histamine–cytokine network [50]. Histamine is involved in the regulation of the expression and activity of cytokines; in turn, the latter affects the level of histamine release from cells [49,51].

Thus, Marone et al. showed that in vitro, an increase in the concentration of histamine in the medium promotes the release of IL-6 and beta-glucuronidase from macrophages isolated from the human lung parenchyma [50]. Histamine induces the production of IL-31, which plays an important role in pruritus and skin barrier function in allergic dermatitis [52].

H2R is present in parietal cells of the stomach, enterocytes, endothelium, vascular smooth muscle cells, sinus node cardiomyocytes, immunocompetent cells (mainly lymphocytes), and muscle plexus ganglia. The main effects of H2R stimulation are external secretion (primarily hydrochloric acid), tachycardia, relaxation of smooth muscle cells, anti-inflammatory effects (suppression of the production of cytokines IL-12, IFN-γ, TNF-α by monocytes or macrophages and mast cells, proliferation of T-helper 1 and type 2, and production of antibodies) [42]. Knockdown of H2R−/− mice demonstrated impaired immune functions, gastric acid secretion, and cognitive functions associated with impaired potentiation of the hippocampus and abnormalities of nociception [53,54,55].

H2R transmits signals in a different way from H1R, namely through cyclic AMP (cAMP) [56]. Interestingly, the activation of H1R and H2R has opposite effects for various biological processes. In a T-cell-mediated immune response, H1R activation promotes Th1 polarization, while H2R activation suppresses Th1 polarization [1,57]. Binding of histamine to the H2R receptor leads to an increase in the production of IL-10 and a decrease in the secretion of IL-12 [57]. As a result, DCs with histamine maturation polarized naive CD4 (+) T cells towards the Th2 phenotype in comparison with immature dendritic cells matured in the absence of histamine. Mazzoni et al. suggested that Th2-mediated IgE production leads to increased secretion of histamine by mast cells, providing a positive feedback loop, exacerbating the pathophysiology of atopic diseases [57].

The opposite effect of H1R and H2R activation has also been observed in smooth muscle contraction. H1R and H2R antagonists, respectively, suppress and exacerbate histamine-induced bronchospasm in patients with mild asthma [58]. These data suggest that histamine may have opposite effects depending on the specific histamine receptor being activated.

H3Rs are located exclusively in the central nervous system (basal ganglia, cortex, hippocampus, and striatum) [42]. H3R mediated by interactions with Gαi/o stimulation reduces the production and release of acetylcholine, serotonin, and norepinephrine. Changes in the functional activity of H3R play an important role in sleep disorders, attention deficit hyperactivity disorder, epilepsy, and cognitive impairment [59]. H3R-deficient mice have shown changes in behavioral responses and movement, as well as metabolic syndrome accompanied by obesity, hyperphagia, and increased levels of leptin and insulin [4,60]. H3R knockout can also lead to increases in the severity of neuroinflammatory diseases and the expression of IFN-inducible protein 10 in T cells [61].

Lieberman showed the role of H3R in inflammation in rhinitis [20]. This is probably due to the fact that it is expressed on presynaptic nerves in the peripheral sympathetic adrenergic system, as well as on the nasal submucosal glands [62]. It is likely that the localization of H3R around the submucosal glands plays an important role in the secretion of the nasal submucous glands.

Histamine is not only a neuromodulator, but also an actor in neuroinflammation and interactions between mast cells, microglia, and astrocytes. Histamine is a promising therapeutic target with potential roles in the prevention or treatment of signs and symptoms of various nervous system developmental disorders (NDDs) [63]; however, there are still no convincing studies substantiating the involvement of mast-cell-secreted histamine in modulating both neuroinflammation and synaptic development and plasticity in vivo. Studying the mechanisms of early modulation of mast cell function is of great importance, since it may provide a new therapeutic target for NDD, including Tourette’s syndrome, autism spectrum disorders, attention deficit hyperactivity disorder, and schizophrenia [63].

H4Rs are mainly present on the immune cells of peripheral blood, leukocytes, and mast cells of the lamina propria and submucosa, spleen, thymus, bone marrow, mucosa-associated lymphoid tissue, as well as in the intestinal epithelium and neuroendocrine cells. They are also found in the bile and pancreatic ducts [42]. According to some reports, H4Rs mediated by interactions with proteins Gα/io are involved in the development of inflammation and hypersensitivity reactions [64,65]. H4R activation significantly enhances the inflammatory response in experimental colitis, radiation colitis, ischemic/reperfusion injury of the intestine, and allergic reactions. In this regard, the development of selective H4R blockers may represent a promising approach for the treatment of inflammatory bowel diseases [66,67]. H4R activation has been established to play a role in the pathogenesis of peptic ulcers and carcinogenesis [65]. Activation of H4R and H3R enhances the effect of acetylcholine on intestinal peristalsis [68].

H4R-mediated mast cell activation results in the expression of proinflammatory cytokines and chemokines IL-6, TNF-α, TGF-β1, RANTES, IL-8, MIP-1α, and MCP-1 [8]. Stimulation of mast cells with histamine H4R promotes mast cell chemotaxis at the site of an allergic reaction [5]. In addition, stimulation of mast cells increases the levels of FcεRI expression and protein presentation on the surfaces of mast cells and promotes degranulation by mobilizing intracellular calcium [8].

Basophils also express H4R on their surfaces and release histamine after antigen stimulation. Histamine, acting through H4R, induces chemotaxis in bone marrow basophils [69].

As previously noted, histamine-specific receptors differ in their tissue localization, function, and affinity for histamine. Histamine regulates a wide range of metabolic processes, having both pro-inflammatory and anti-inflammatory effects, depending on the receptor subtype and the type of stimulated cells [8]. The various effects of histamine on immune regulation appear to be related to the differential expression and regulation of its receptors and their individual intracellular signals. In addition, differences in the affinity of these receptors for histamine play a critical role in the biological effects of histamine and drug ligands for histamine receptors [39]. Histamine receptors play an important role in various pathophysiological conditions and are effective targets for the treatment of allergies; however, the mechanism of receptor activation remains unclear [46].

## 4. Histamine Intoxication

Histamine and related biogenic amines (tyramine and putrescine) enter the body with food. Their content is especially high in high-protein nutrients (fish or meat) that are stored in unsuitable conditions or significantly contaminated with microorganisms. The enzyme histidine decarboxylase, which catalyzes the formation of histamine from the amino acid histidine, remains active even at relatively low temperatures and is quickly reactivated after thawing. Direct contact of fish and meat with bacteria occurs during cutting, chopping, and mixing with vegetables. Some histamine-forming bacteria are resistant to table salt and are even halophilic, which means they actively produce histamine when salted, smoked, or dried. A number of histamine-generating bacteria are facultative anaerobes that remain viable in vacuum packaging. Histamine accumulation is quite resistant to high and low temperatures [70]. Approximately 40% of the toxic reactions associated with the consumption of fish and seafood are precisely caused by the action of biogenic amines. Because such cases were more often recorded with fish of the suborder Scombridae (mackerel, tuna), the term “scombroid poisoning” was previously used. The World Health Organization (WHO) recommends labeling this condition as “histamine intoxication” [71]. The corresponding symptoms appear after a few minutes each hour and include perspiration, burning in the mouth and throat, urticaria and itching, arterial hypotension, tachycardia, headache and dizziness, nausea, vomiting, diarrhea, and bronchial obstruction [71,72]. An increased content of histamine and serotonin is noted in fermentation products (cheeses, wine, canned food, pickled vegetables, and some drinks). Lactic acid bacteria (Lactobacillus hilgardii, L. buchnerii, L. curvatus, and Oenococcus oeni) and some strains of enterobacteria can be important sources of histamine [71,72]. A meta-analysis of scientific reports revealed that in 98% of cases, the cause of histamine intoxication was the consumption of fish and seafood, while the remaining 2% were due to cheese consumption [73].

## 5. Syndrome of Histamine Intolerance

This term refers to a situation in which the enhanced biological effects of histamine are observed with the consumption of foods containing this substance. Unlike histamine intoxication, the severity of which is directly proportional to the content of histamine in nutrients, the pathogenesis of histamine intolerance (HI) is associated with congenital or acquired deficiency of enzymes that neutralize histamine. First, we are referring to a decrease in the activity of the “barrier” enzyme, DAO. Experimental suppression of DAO is accompanied by the development of anaphylaxis in response to the oral administration of a physiological dose of histamine. Acquired DAO deficiency may be due to pharmacological effects or intestinal diseases, in particular lactase deficiency, celiac disease, and inflammatory diseases [24]. In intestinal diseases, the degree of DAO deficiency directly correlates with the severity of mucosal damage, intestinal permeability, and malabsorption of carbohydrates [24,74,75,76].

The development of HI is also facilitated by substances and drugs that affect the metabolism of histamine (for example, alcohol and monoamine oxidase inhibitors). Approximately 20% of Europeans regularly take drugs that reduce DAO activity, which increase the risk of developing HI; these drugs include verapamil, clavulanic acid, chloroquine derivatives, acetylcysteine, amitriptyline, metamizole, and isoniazid. Alcohol and acetaldehyde increase the release of endogenous histamine and competitively interact with aldehyde dehydrogenase, reducing the rate of histamine degradation [17,24,77].

Deficiencies of copper, vitamin C, and pyridoxine may be possible reasons for the decreased activity of DAO [17]. DAO activity also depends on the phase of the menstrual cycle [24].

Polymorphisms in the coding gene may cause congenital deficiency (in particular, rs10156191, rs1049742, and rs2268999), or in contrast may cause an increase of DAO activity (rs2071514, rs1049748, and rs2071517) [78].

An important role in the pathogenesis of HI is assigned to the features of the intestinal microbiota and dysbiosis inherent in diseases of the digestive system. Decarboxylation reactions, which are essential for histamine production, are considered a survival strategy for microorganisms due to decreases in pH and are an alternative source of energy. Bacterial DAO is involved in the production of ammonia and hydrogen peroxide. In patients with signs of HI, increased amounts of Proteobacteria and Roseburia in feces, significant decreases in the α-diversity of the intestinal microbiota, and increased levels of zonulin were found, which are considered indicators of increased intestinal barrier permeability [79].

The clinical manifestations of HI are diverse and non-specific, as explained by the specific distribution of the four types of histamine receptors in organs and tissues. The most frequent and pronounced manifestations are gastrointestinal, including bloating (92% of cases), feeling of fullness after eating, diarrhea, abdominal pain, and constipation (55–73% of cases). The second most frequent manifestations are disorders of the nervous and cardiovascular systems (dizziness, headaches, and palpitations) and respiratory and dermatological symptoms. Combinations of three or more gastroenterological symptoms with damage to other organs were reported in 97% of cases (on average, 11 symptoms in one patient) [80]. HI may underlie the development of atopic eczema and migraine, as well as other vascular reactions triggered by certain foods [81,82].

According to the 2003 recommendations of the World Allergy Organization, non-immunological pathological reactions to food should be classified as “non-allergic food intolerance” [83]. The prevalence of HI is estimated to be 1–3% [24]. Insufficient specificity and variability in response to histamine complicate the development of clear diagnostic criteria for this phenomenon; however, it is suggested that the following algorithm be followed.

Stage 1: In the presence of ≥2 clinical symptoms characteristic of HI, IgE should be excluded—mediated allergic reactions (using skin tests) and systemic mastocytosis (study of serum tryptase activity) and concomitant diseases of the digestive system should be evaluated, while medical history (taking medications that inhibit DAO) should be assessed.

Stage 2: For 4–8 weeks, patients should adhere to a diet low in histamine, registering the dynamics of all existing symptoms. The clinical component of HI should be decreased in the presence of HI.

Stage 3: Laboratory diagnostics should be performed, including determination of DAO activity in blood serum samples or intestinal biopsy, oral histamine challenge test, skin prick test with histamine (assessment within 50 min), research of genetic polymorphisms of DAO, study of the content of histamine metabolites (methylhistamine) in urine and feces.

The “barrier enzyme”, DAO, limits the flow of histamine from the intestinal lumen into the internal environment. The threshold value of serum DAO activity, below which the likelihood of HI is high, is 10 U/mL; however, DAO activity in the same person is highly variable. Perhaps this is why in some cases, when registering low DAO activity, there are no clinical symptoms, while when the clinical signs of HI are present, DAO activity indices correspond to the norm. In addition, the origin of HI may be due to other reasons [24,84].

A histamine skin prick test may not be helpful if there is acquired DAO deficiency in the presence of intestinal disease. In such situations, an oral histamine challenge test should be used and urinary histamine metabolites should be examined. Oral histamine challenge carries the risk of serious side effects and should only be performed in a hospital setting. In addition, a universal protocol for this test has not been developed [24].

It is noteworthy that the oral administration of “pure” histamine in doses equivalent to those contained in foods, which provoke the development of HI symptoms, does not allow for the reproduction of symptoms of the same range or severity. Alcohol and biogenic amines (putrescine, cadaverine, and tyramine) present in nutrients may, thus, have potentiating effects [24].

HI must first be differentiated from IgE-mediated hypersensitivity to food components (“food allergy”), celiac disease, hypersensitivity to nickel, various diseases associated with diarrhea, and conditions associated with gastric hypersecretion [24]. To exclude IgE-mediated hypersensitivity, elimination diets, skin tests, or the determination of specific IgE to the most common food allergens (milk, eggs, fish, shrimp, soy, nuts) are recommended. To exclude celiac disease, a study of antibodies against tissue transglutaminase was performed. The detection of hypersensitivity to nickel and other metals is based on historical data and the results of the application test [24,84].

The clinical picture of HI is similar to that of mast cell activation syndrome, which manifests as episodes of intestinal and systemic symptoms caused by degranulation of mast cells. They can be recurrent or persistent and depend on the eating pattern. Reliable diagnostic markers have not yet been developed [85]. Unlike clonal activation (e.g., in mastocytosis), mast cell activation syndrome is characterized by normal basophils and mast cells, which may increase in response to a trigger (“reactive hyperplasia”). The triggers can be IgE complexes with various ligands, including allergen, drugs (non-steroidal anti-inflammatory drugs, vancomycin), complement components, aberrant IFNγ, and infectious agents acting on toll-like receptors [86,87].

## 6. Mast Cells in Bowel Disease

Mast cells are found in mucous and epithelial tissues throughout the body and are located at the interface between the host and the external environment at the sites of antigen penetration (gastrointestinal tract, skin, respiratory epithelium) [88]. The mast cell cytoplasm contains 50–200 large granules that store inflammatory mediators, including histamine, tryptase, serotonin, interleukins-1 and -6, platelet activating factor, prostaglandins, and leukotrienes [89]. Mast cells are the main sources of endogenous histamine, the release of which can be rapid and massive (“anaphylactic degranulation”) or only partial (“marginal degranulation”).

Using immunohistochemical methods, two types of human mast cells have been identified in the skin, lungs, and small intestine [90]. Mucosal mast cells, which produce only tryptase (T-type), and connective tissue mast cells, which produce chymase, tryptase, and carboxypeptidases (TC-type) [90,91].

In the gastrointestinal tract, mast cells are concentrated mainly in the lamina and submucosal layer at the border of the external and internal environment. Further, they are present in small amounts in the epithelium, muscles, and serous layers (T-type) [92]. T-type mast cells (containing tryptase) predominate in the mucous membranes of the intestines and lungs. The skin and lymph nodes are dominated by TC-type mast cells (containing tryptase, chymase, and carboxypeptidase) [93].

Mast cell activation and neurotransmitter release affect tissues and organs in different ways. The mucous membrane of the respiratory tract, the gastrointestinal tract, and the skin are most often exposed to the actions of antigens [94]. The T-type of mast cells in the skin accounts for 12%, while the type found in the mucous membrane accounts for 98% [90]. In the lungs, T-type mast cells are predominant (up to 93%); however, enzyme activity examination showed a minor presence of chymase activity in T cells [95], whereas the foreskin is characterized by the prevalence (up to 99%) of chymase TC mast cells. It should be noted that wherever cells are located in the body, the tryptases in both T and TC mast cells are identical in their ultrastructure and contain identical subunit compositions and specific activity properties according to Western blot analysis [96,97].

The physiological and pathobiological consequences of T and TC types of human mast cells, characterized by different mediators, remain to be studied [88,98]. Tryptase, which is present in both T- and TK-type mast cells, generates anaphylatoxin C3a (a factor of the complement system) and acts as an anticoagulant, inactivating high molecular weight kininogen and fibrinogen [92]. In turn, chymotriptin (TC-type) in lung mast cells is a potent activator of angiotensin I for angiotensin II [90]. Carboxypeptidase A3 is expressed in the same granules that contain tryptase and chymase (TC-type) [99]. Carboxypeptidase A3, as with chymase, is released as a complex with proteoglycans [100]. In experiments on mice, co-expression of carboxypeptidase and chymase [101] has been shown.

Mast cells are involved in the reactions of innate and acquired immunity. The classic stimulus causing massive degranulation of mast cells is the interaction of IgE with the FcRI receptor and allergen (“anaphylactic degranulation”). Mast cells also express IgG receptors, other immunoglobulin receptors, complement receptors, and pattern recognition receptors (in particular, Toll-like receptors). Interactions with appropriate ligands (for example, in infectious diseases) are usually accompanied by marginal degranulation of these cells. It is extremely difficult to estimate the volume of secretion released from granules. In addition, marginal degranulation is possible under the influence of hormones, neurotransmitters (acetylcholine, serotonin, neuropeptides, and factors released from nerve endings, immunocompetent cells produced by the microbiota), and certain drugs (fluoroquinolones). Mast cells closely interact with the enteric nervous system, as well as sensory C-fibers of the vagus nerve and spinal afferent fibers, which express type 1 vanilloid receptors (TRPV1) [92,102]. In healthy individuals, the excitability of TRPV1-expressing neurons increases after incubation with histamine, and this effect is blocked by H1R antagonists [93]. Mast cells express corticotropin-releasing hormone receptors 1 and 2 and degranulation can occur when corticotropin-releasing factor is released by eosinophils or CNS cells (for example, under stress) [93].

The intestinal microbiota and functional state of the intestinal nerves have a significant effect on the functional state of mast cells. Bacterial infection causes degranulation of mast cells and the release of mediators [103,104]. Increases in histamine and tryptase secretion have been reported in intestinal biopsies from patients with IBD [105]. These subjects show a lower bacterial diversity of the gut microbiota, an increase in the Proteobacteria type, and a decrease in Firmicutes [103]. Mast cells are activated and secrete chymase and matrix metalloprotease-9, which increase the permeability of the mucous membrane. The secretion of IL-1β, IL-6, and TNF-α by T-type mast cells creates the basis for the persistence of inflammation, while the secretion of histamine and tryptase contributes to the sensitization of nerve fibers [102].

The assessment of mast cell activity is complex and ambiguous. Previously, Giemsa or toluidine blue staining were used to visualize these cells. Currently, a more sensitive and specific immunohistochemical method is utilized to identify the surface c-kit receptor [CD117] or cytoplasmic tryptase. To exclude systemic mastocytosis, a receptor with low affinity for IL-2 (CD25) was investigated [106].

The physiological function of mast cells is to serve as a rapid signal of the presence of damaging factors, as well as cleanse the intestinal lumen by stimulating secretion and peristalsis. Histamine, chymase, and prostaglandin D2 increase the secretion of chloride and water and induce segmental contractions a second time (TC-type). The vagus nerve can modulate the functional state of mast cells (“neuroimmune interaction”) [92,107].

Excessive number and activity of mast cells contribute to the development and maintenance of inflammation in the gastrointestinal tract (functional and inflammatory diseases, food allergies, postoperative intestinal obstruction, and malignant tumors). The number of mast cells in the intestine is increased in diseases accompanied by abdominal pain and postprandial diarrhea. Consequently, an excess of histamine is important in the pathogenesis of intestinal and extraintestinal symptoms [102,105,108].

In a significant number of patients with functional dyspepsia and irritable bowel syndrome with a predominance of diarrhea (IBS-D), hyperplasia and the increased activity of mast cells were revealed, which are associated with increased excitability of neurons and intestinal permeability [92,109,110,111,112,113]. Hyperplasia in IBS-D was noted not only in the colon and terminal ileum, but also in the duodenum and jejunum. Perhaps the most important factor is not the number of mast cells, but their activity [114]. The number of mast cells located in the immediate vicinity (5 µm) of nerve fibers directly correlates with the intensity and frequency of abdominal pain [92,115]. In IBS-D, the expression of H1R and H2R is increased in the intestinal tissues. Simultaneously, H4R expression is not impaired. The significance of these receptors has been insufficiently studied, although their important role in the regulation of visceral sensitivity and intestinal peristalsis is known [42]. Colon biopsies with IBS-D showed an increase in tryptase activity, which directly correlated with the activity of nuclear factor kB (NF-kB), initiating the inflammatory reaction in response to bacterial or viral components, oxidative stress, and proinflammatory cytokines [116]. The administration of H1R, H2R, and H3R blockers to patients with IBS-D led to a decrease in the hyperexcitability of neurons in the submucosa of the colon [117,118].

In IBS-D, hyperexcitability, mediated by the action of histamine on H1R, was noted for submucous neurons and for TRPV1 neurons of the ganglia of the dorsal roots of the spinal cord [93].

Intestinal symptoms occur or worsen after ingestion of certain foods in more than half of patients with IBS-D. Symptoms appear within 15 min in 28% of patients and within 3 h in 93% of patients. Food triggers (histamine liberators) are foods rich in biogenic amines (canned food, wine, beer, and cheeses), as well as alcohol. Food intolerance can also be realized through the interaction of food antigens with IgG, followed by marginal degranulation of mast cells. The elimination diet was found to result in clinical improvement in over 70% of the cases.

The systemic vasomotor reactions observed in some patients with IBS-D are due to the influence of histamine. In response to the intake of H1R blockers, these patients have an increase in the average daily systolic blood pressure, which may indirectly indicate the probable contribution of histamine to disorders of vascular regulation in functional diseases of the digestive system [119].

Meanwhile, the mast cells of the gastrointestinal tract and the histamine they produce have an anticarcinogenic effect. It has been experimentally established that the deficiency of bacterial histidine decarboxylase promotes the development of inflammation and tumors in the intestine, combined with a defect in myelopoiesis and overproduction of pro-inflammatory cytokines. Administration of histamine-producing L. reuteri promotes the regression of these disorders [30].

## 7. Principles of Treatment for Histamine Intolerance

The foundation of histamine intolerance (HI) therapy is an elimination diet based on foods with low histamine content. Generally, accepted dietary guidelines have not been developed; however, it is recommended to avoid the consumption of hard and semi-hard cheeses, oily fish, and shellfish in any form, as well as raw fermented meat products, pickled vegetables, fermented soy products, wine and beer, chicken eggs, chocolate, and mushrooms. Meat and fish can only be eaten if fresh. In addition, vegetables and fruits that stimulate the release of endogenous histamine (such as spinach, tomatoes, citrus fruits, strawberries, eggplant, avocado, papaya, bananas, kiwi, pineapples, and plums) should be avoided. DAO-containing food additives based on enteric-coated pig kidney extracts have been developed. Legume sprouts are also used as a source of DAO, in which the activity of this enzyme is 250-fold higher than that in non-sprouted seeds. The effectiveness of the elimination diet has been proven in clinical studies. After 4 weeks of appropriate nutrition, decreased severity of gastroenterological and dermatological symptoms, as well as migraine abolition, was noted in 33–100% of cases [73,77,81]. With regard to diet, some patients showed an increase in the plasma activity of DAO [4]. Supplementation with DAO was also found to improve the quality of life of patients with HI [120].

HI can be combined with gluten intolerance and disaccharidase deficiency. In this case, an elimination diet with restriction of fermentable oligosaccharides, disaccharides, polyols, and gluten is shown [121].

Medication schemes for HI correction are based on the use of histamine receptor blockers, mast cell membrane stabilizers, and other drugs with antihistamine activity. In this regard, H1R antagonists are the most effective, particularly ebastine (Kestin^®^). Taking this drug at a daily dose of 20 mg for 12 weeks resulted in clinical remission in 46% of patients with IBS-D; however, H2R blockers were found to be ineffective in individuals with HI [93,114,122].

Mast cell membrane stabilizers (disodium cromoglycate and ketotifen) compete with H1R blockers in terms of their effectiveness. Cromoglycate disodium at a daily dose of 800 mg after 4 weeks of administration significantly reduced abdominal pain and diarrhea in 30–40% of patients with functional dyspepsia syndrome and IBS-D. This drug was found to prevent an increase in intestinal permeability under conditions of acute psychological stress [123,124].

Ketotifen has a wide range of actions. As a stabilizer of mast cell membranes, it blocks H1R, and to a lesser extent H4R, and inhibits phosphodiesterase and leukotrienes. At a daily dose of 2 mg, after 4 weeks of administration, this drug resulted in clinical remission in approximately 50% of patients with IBS-D, with a treatment duration of 4–6 months [125]. It has been established that ketotifen prevents chemically induced colitis and reduces the degree of inflammatory activity [67].

Mirtazapine (Remeron^®^), an antidepressant from the group of type 3 serotonin receptor antagonists, which also has antihistamine activity, is widely used to treat patients with HI and IBS-D. A daily dose of 30–60 mg after 3 weeks of administration was found to eliminate stool disorders and abdominal pain in approximately 60% of cases. The course of treatment was 4–6 months [126].

Vitamin C was used as an adjuvant to correct the HI. A daily dose of 300–500 mg enhanced histamine degradation and inhibited mast cell degranulation [127]. Natural flavonoids (fisetin, kaempferol, quercetin, rutin, and luteolin) and the active alkaloid berberine inhibit mast cell degranulation in vitro [128].

Clinical care for HI patients is usually limited to a low-histamine diet [24]. Oral supplementation with the exogenous enzyme DAO from pig kidneys is also used to increase the gut’s ability to eliminate histamine from food. Research is underway to identify new sources of the DAO enzyme, including plant [24]; however, additional interdisciplinary HI studies integrating food science, physiology, and biochemistry are warranted, as well as clinical trials aimed at extending available diagnostic approaches and strategies for the treatment of histamine intolerance.

## 8. Conclusions

Histamine is an important regulator of intestinal homeostasis. By enhancing secretion and peristalsis, histamine promotes the rapid removal of potentially toxic substances. This biogenic amine is also essential for the survival of gut bacteria; however, its excessive accumulation due to congenital or acquired deficiency of the DAO enzyme, as well as hyperplasia and hyperactivity of intestinal mast cells, causes diarrhea and abdominal pain.

The non-specificity of HI symptoms and the absence of verified diagnostic tests significantly complicate the differential diagnosis of intestinal diseases that occur with stool disorder and abdominal pain. The presence of systemic manifestations of HI (in particular vascular reactions and bronchial obstruction) encourages patients to seek medical attention from a different specialization. In this regard, the method used for differential diagnostic searches for suspected HI should include: (1) maintenance of a food diary for 2–4 weeks to identify a causal relationship between the occurrence of the corresponding symptomatology and the use of certain nutrients; (2) exclusion of food IgE-mediated allergies (skin prick test and specific IgE study), celiac disease (antibodies to gliadin, endomysia, and tissue transglutaminase), and inflammatory bowel diseases (analysis for calprotectin, colonoscopy).

Modern treatments for histamine intolerance include an elimination diet, drugs that affect histamine levels (H1R blockers, mast cell membrane stabilizers, and mirtazapine), and natural ingredients (vitamin C, flavonoids, DAO-fortified supplements, and probiotics based on *L. faecalis*).

The important roles of mast cells and histamine in the pathogenesis of many gastroenterological diseases gives rise to an urgent need for further scientific research in this field.

## Data Availability

This is a review paper that collected from public data listed in the “Reference”.
